# Thermal Treatment of Aerosol Deposited NiMn_2_O_4_ NTC Thermistors for Improved Aging Stability

**DOI:** 10.3390/s18113982

**Published:** 2018-11-15

**Authors:** Michaela Schubert, Christian Münch, Sophie Schuurman, Véronique Poulain, Jaroslaw Kita, Ralf Moos

**Affiliations:** 1 Department of Functional Materials, Universität Bayreuth, 95440 Bayreuth, Germany; functional.materials@uni-bayreuth.de (M.S.); functional.materials@uni-bayreuth.de (J.K.); 2 Vishay Electronic GmbH, Dr.-Felix-Zandman-Platz 1, 95100 Selb, Germany; CC-NLR-Division@vishay.com; 3 Vishay Resistors Belgium BVBA, Twee Huizenstraat 37, 1140 Brussel, Evere, Belgium; CC-NLR-Division@vishay.com (S.S.); CC-NLR-Division@vishay.com (V.P.)

**Keywords:** aerosol deposition method (ADM), room temperature impact consolidation (RTIC), spinel ceramic, non-linear resistor, temperature sensor, long-term stability, NTCR

## Abstract

This paper examines the influence of a short-term thermal treatment of aerosol deposited negative temperature coefficient (NTC) thermistor films on the NTCR characteristics and their long-term stability with different electrode materials. An aerosol deposition of a spinel-based NiMn_2_O_4_ powder on alumina substrates with screen-printed AgPd and Au interdigital electrode structures was performed. The manufactured components of the typical size of 1206 were tempered in a moderate temperature range of 200 °C to 800 °C and aged for 1000 h at 125 °C in air. Based on *R*-*T* measurements in a high-precision silicone oil thermostat bath and high temperature XRD analyses, the influence of the thermal treatment was analyzed and discussed. A 60-min tempering at 400 °C proved to be optimal, as both the NTCR parameters and their ageing stability could be significantly improved. The findings are explained.

## 1. Introduction

Spinel-type nickel manganite Ni*_x_*Mn_3-*x*_O_4_, such as NiMn_2_O_4_ (*x* = 1) are semiconducting ceramics, the electrical resistance of which decreases approximately exponentially with increasing temperature (thermally sensitive resistor, thermistor [1]). Due to their high negative temperature coefficient (NTC), nickel manganites are a well-known and a widely used base materials class for ceramic NTC thermistors in industry [2,3]. They are characterized by low costs and high reliability and are used in particular for temperature measurement and compensation [4,5,6]. Their high temperature dependence of the electrical resistance is the result of a small polaron hopping-based conductivity mechanism [7] and can be simplified by the Arrhenius relationship:

*R*(*T*) = *R*_0_ exp(*E*_A_/(*k*_B_·*T*)) (1)

where *R*_0_ is the resistivity at an infinite temperature, *E*_A_ the activation energy for the hopping process, *k*_B_ the Boltzmann constant and *T* the absolute temperature [8,9]. The quotient of *E*_A_ and *k*_B_ is defined as the *B* constant, a characteristic parameter for NTCR ceramics. Today, NTCR sensors are mainly bulk ceramics [10], which are produced in the form of chips, discs or SMD [11]. They are usually produced by traditional ceramic and sinter-based processes with controlled sintering profiles and temperatures above 1000 °C [12]. These processes are not very flexible and very labor- and energy-intensive. Due to the poor sinterability of NTCR ceramics, problems such as porosity, lack of stability and reproducibility must also be handled [10,13,14].

A new approach to produce film-based NTCR devices utilizes the Aerosol Deposition Method (ADM) [15,16,17,18,19,20]. With this novel spray coating process, dense ceramic NTCR films can be produced at room temperature and directly from a ceramic spinel-based starting powder on various substrate materials [21,22]. The deposited NTCR films are characterized by a nanocrystalline film structure, a high density, good substrate adhesion and a film thickness between 0.5 µm and 5 µm [15,16,17,18,19,20]. The electrical properties of the NTCR films are similar to those of the traditionally produced bulk-based NTCR ceramics and can be improved by moderate film tempering up to 600 °C [19]. Although much progress has been made in the field of aerosol deposition of NTCR films, some questions still need to be answered before the process can be used for commercial NTCR production. In addition to the reproducibility of the NTCR characteristics (resistance at 25 °C, *R*_25_; specific resistance at 25 °C, *ρ*_25_; and the *B* constant), long-term stability also plays a decisive role. It is known from the field of NTCR ceramics that most thermistor ceramics undergo a change (usually an increase) in electrical resistance over time and under thermal stress, which is referred to as aging [23,24,25]. The degree of aging or “aging resistance” is determined by the composition and the thermal history. Especially the temperature, the atmosphere, and the cooling rate during sintering, but also during subsequent electrode firing (e.g., ‘serigraphy’, a process commonly used in industry), have been found playing a decisive role [6,24,26,27]. While the aging resistance of bulk ceramics decreases due to thermal post-treatment (e.g., by metallization at 850 °C [6]), the aging resistance of thin films is improved by subsequent tempering up to 750 °C [28]. To what extent thermal treatment affects the aging resistance of aerosol-deposited thick films and whether there are interactions with the electrodes will be investigated in this paper.

## 2. Materials and Methods

**Figure 1:**
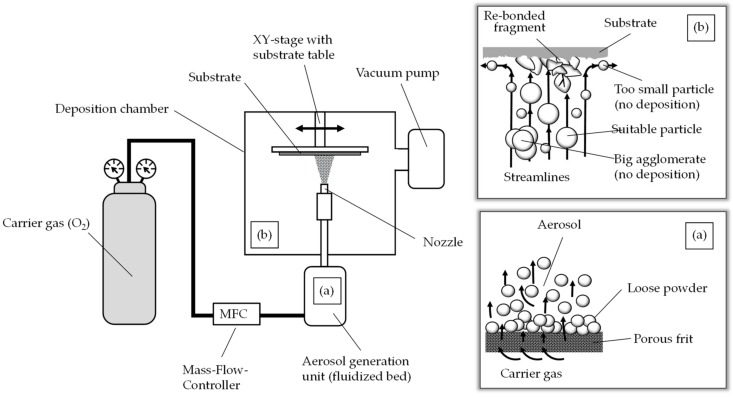
Schematic diagram of an aerosol deposition apparatus and the functional principle: (**a**) aerosol generation by fluidized bed; (**b**) film formation process.

The starting powder for the aerosol deposition is a cubic NiMn_2_O_4_ spinel. The powders were prepared via the classical mixed-oxide route, using commercially available cubic NiO and α-Mn_2_O_3_ powder. The raw oxides were simultaneously mixed and milled in a rotary ball mill in deionized water at 400 min^−1^ for 30 min. Milling bowls and balls (diameter 10 mm) were both made of stabilized zirconium dioxide. The powder mixtures were air dried and calcined in an electric oven at 900 °C for 3 h. The spinel-based powders were then milled again at 400 min^−1^ for 30 min to reduce the particle size. Again, a rotary ball mill with zirconium dioxide milling bowls and balls (diameter 10 mm) was used. To achieve a better deposition behavior of the powder, the powder was dried in a convection oven at 200 °C and sieved with a 90 µm sieve. This breaks up large agglomerates and improves aerosol generation and film formation [29,30]. The prepared NiMn_2_O_4_ powders were then deposited by ADM on aluminum oxide substrates (Rubalit 710, CeramTec) that were provided with screen-printed interdigital electrode structures. To investigate the influence of the electrode material on aging, the screen-printed electrodes were made of gold Au (5744R, DuPont) and silver palladium AgPd (6146, DuPont). In the case of Au electrodes, the interdigital electrode structure consisted of nine pairs of electrode fingers with a finger line/space of 70 µm. The AgPd interdigital electrode structure consisted of six finger pairs with a finger line/space of 100 µm. During the deposition process, the screen-printed contact pads were covered with a paper-based disposable mask. The aerosol deposition was carried out in a custom-build aerosol deposition device, which is described e.g., in [31,32] and shown schematically in Figure 1. Typically, an apparatus for the aerosol deposition consist of an aerosol generation unit, a deposition chamber, a nozzle and a vacuum pump. There are various possibilities for aerosol generation [33,34]. In this paper, an aerosol generation system based on a fluidized bed was used. As shown in Figure 1a, the spinel-based starting powder is located on a porous frit. A carrier gas, here oxygen with a flow rate of 6 L/min, is passed through the loose powder. Powder particles are entrained and an aerosol is generated. The flow rate of the carrier gas is adjusted via a conventional mass flow controller. Due to the pressure difference between the aerosol generation unit (~20 kPa) and the deposition chamber (~0.5 kPa), the aerosol is transported into the deposition chamber. 

**Figure 2:**
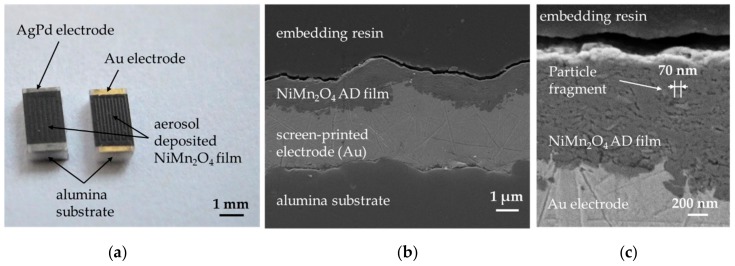
(**a**) Aerosol deposited NiMn_2_O_4_ film on alumina substrates with screen printed AgPd electrodes (left) and Au electrodes (right); (**b**) Cross section of a NTCR component in SEM, consisting of a NiMn_2_O_4_ AD film on screen-printed Au electrodes on alumina substrate; (**c**) Detailed view of (**b**).

On its way into the deposition chamber, the aerosol passes through the nozzle (here a 0.5 mm × 10.0 mm slot nozzle). The change in cross-section accelerates the aerosol to several hundred meters per second. The aerosol jet is directed at a substrate that is positioned at a small distance (here 4 mm) perpendicular to the nozzle on a substrate holder. Suitable particles collide with the substrate, while too large agglomerates, too large particles and too small particles are not deposited and remain in the chamber as overflow (Figure 2b, details in [21]). The impact of a suitable particle on the substrate probably leads to a local increase in pressure and temperature [35] as well as to a plastic deformation and/or fracture [21] of the particle in fragments. The surfaces of the particle fragments are reactive and form new connections with the substrate as well as with each other. This leads to film formation. The process begins with the formation of an anchor layer and continues with the formation and densification of the film by a hammering effect of the subsequently impinging particles [36]. This process is often referred to as “Room Temperature Impact Consolidation” (RTIC) [22]. By a horizontal movement of the substrate holder through an XY table at low speed, in this case 1 mm/s, a broad area is covered with the film. After successful deposition, the paper-based disposable mask was removed and the components with the typical size of 1206 (3.2 mm × 1.6 mm) were separated along the laser-cut break edges. 

The NTCR components were analyzed via SEM (Leo 1450 VP, Zeiss, Oberkochen, Germany). The crystal structure was determined by XRD (PANalytical XPert Pro, PANalytical, Almelo, The Netherlands) both of the starting powder and of the aerosol film on a Si wafer in the deposited and tempered state (after 30 min at 200 °C, 400 °C, 600 °C, 800 °C, respectively, in air). Crystallite size and internal stress were determined using the Williamson–Hall method [37]. For this purpose, the reflex broadening in half of the reflex maximum Δ(2*Θ*) in radians was determined using the Lorentz fit function on reflexes (111), (220), (311), (222) and (400). By fitting the Williamson–Hall equation (see Equation (2)), the crystallite size *L* and the internal stress *ε* were determined:

Δ(2*Θ*)*·*cos(*Θ*) = (*K·λ*)/(*L*) + 4 · *ε·*sin(*Θ*) (2)

where *K* is the dimensionless Scherrer constant with 0.9, *λ* is the wavelength of the X-ray source (here Cu Kα1: 1.5406 Å) and *Θ* is the Bragg angle in degree. The devices were electrically characterized in the deposited, in the moderately tempered, and in the aged state. For the electrical characterization, the electrical resistance was measured at 25 °C and at 85 °C and the *B* constant as well as the specific resistance at 25 °C *ρ*_25_ were calculated. *R*_25_ and *R*_85_ were measured in a high-precision thermostatic bath (Julabo SL-12, Julabo GmbH, Seelbach, Germany) with silicone oil (DOW CORNING^®^ 200 FLUID, 5 CST, Dow Corning Corporation, Midland, MI, USA) using a digital multimeter (Keithley 2700, Keithley Instruments Inc., Solon, OH, USA). The temperature was controlled using a high precision Pt1000. The *B* constants were calculated, using Equation (3) [9]:

*B* = ((*T*_25_·*T*_85_)/(*T*_85_ − *T*_25_))·ln(*R*_25_/*R*_85_) (3)

thereby *T*_25_, *T*_85_ are temperatures of 25 °C and 85 °C expressed in Kelvin and *R*_25_ and *R*_85_ are the resistance at 25 °C and 85 °C, respectively. To check the thermal stability of the AD-NiMn_2_O_4_ films, the devices were tempered in an electric furnace with five tempering cycles C800, C600, C400, C200 and H800. All tempering cycles alternately consist of the actual tempering in an electric furnace and an electrical characterization in silicone oil between 25 °C and 85 °C. All tempering steps are carried out in air with a heating/cooling rate of 10 K/min and a dwell time of 60 min. The components are tempered at 800 °C, 600 °C, 400 °C, and 200 °C, respectively, in the C800 tempering cycle. For the tempering cycle C600, tempering takes place at 600 °C, 400 °C, and 200 °C, respectively. The tempering cycle C400 consists of tempering at 400 °C and 200 °C and the cycle C200 has only one tempering step at 200 °C. The tempering cycle H800 examines the tempering behavior with increasing tempering temperature. Consequently, tempering takes place at 200 °C, then is followed by tempering at 400 °C, 600 °C, and 800 °C. Thus, it represents the inversion of cycle C800. In order to test the aging behavior and the influence of the heat treatment on the long-term behavior, the aerosol deposited-NiMn_2_O_4_ film components were aged in the various tempered states. The aging took place in an electric furnace at 125 °C for 1000 h in air. After 24 h, 100 h, 300 h, 500 h, 700 h, and 1000 h, respectively, the components were characterized in the thermostat bath between 25 °C and 85 °C.

## 3. Results

### 3.1. Film Characterization in Deposited State

Figure 2a shows the finished NTCR components with the typical component size of 1206 (1.6 mm × 3.2 mm). The components consist of an alumina substrate, a screen-printed AgPd interdigital electrode (left side) and an Au interdigital electrode (right side) as well as the NiMn_2_O_4_ film deposited thereon.

**Figure 3:**
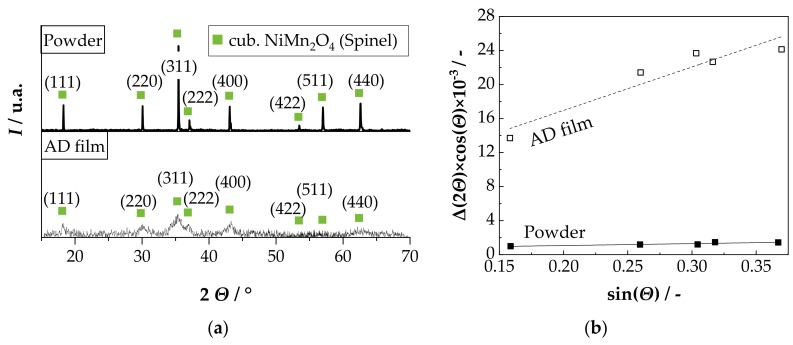
(**a**) Comparison of the XRD patterns of the starting powder and an aerosol deposited film in the as-deposited state; (**b**) Williamson–Hall plot of the starting powder and the AD film in the as-deposited state.

The films are homogeneous and scratch-resistant. In both cases, disposable masking of the contact pads and delamination-free separation in 1206 devices was successful. Figure 2b shows the cross-section of an NTCR component with Au electrode. The adhesion of the screen-printed electrode to the alumina substrate and the adhesion of the aerosol deposited film to the electrode and to the substrate is excellent. The aerosol deposited film has a thickness of approximately 1 µm and is completely dense, without visible pores or cracks. In addition, the fragmented and plastically deformed particles can be seen in the nanometer range in the aerosol-deposited NiMn_2_O_4_ film (see Figure 2c). Also, a kind of layer-like structure of the film can be seen. Thus, a successful film formation via the RTIC mechanism [22] can be assumed. To investigate the influence of the aerosol deposition process on the crystal structure, the XRD pattern of the starting powder was compared with that of the deposited film on the Si wafer at room temperature (Figure 3a). In both XRD patterns only the reflexes of the cubic NiMn_2_O_4_ spinel could be found. No phase change occurred as a result of the deposition process. The reflexes in the aerosol deposited film are much broader and less intensive. This can be explained by the nanocrystalline film structure and the internal strain and is typical for aerosol deposited films [17,32,38]. To determine the crystallite size and the internal strain in the deposited film in comparison to the starting powder, both spectra were examined using the Williamson-Hall method (see Figure 3b).

The evaluation of the data from Figure 3b yields a crystallite size of 225 nm and a negligible internal strain of 0.06% for the starting powder. In comparison, the AD film has a crystallite size of 21 nm and an internal strain of 1.28% in the as-deposited state. The crystallite size of approximately 20 nm corresponds to the results of the SEM analysis in Figure 2b and is typical for aerosol deposited films in the as-deposited state [19,39,40,41,42,43]. The occurrence of noticeable internal strains that stem from the deposition process is also typical [29]. 

**Figure 4:**
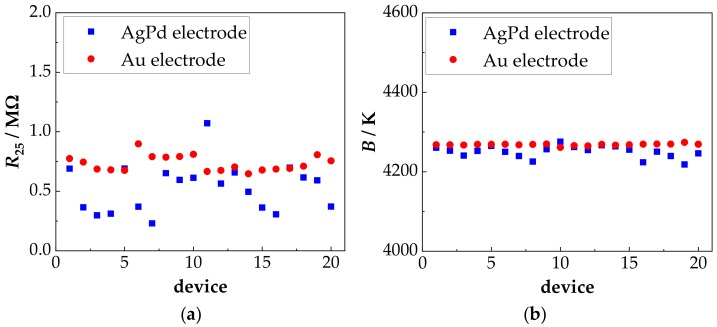
(**a**) *R*_25_ value and (**b**) *B* value of 20 devices with AgPd electrode and 20 devices with Au electrode in the as-deposited state.

The result of the electrical characterization of all NTCR components in the as-deposited state as they were investigated in this work is summarized in Figure 4.

Figure 4a shows the measured resistance values at 25 °C (*R*_25_). For each electrode material (AgPd and Au) 20 resistors were measured. The *R*_25_ values varied from 250 kΩ to 750 kΩ. This scattering is mainly caused by inhomogeneities in the film thickness and by the tolerances of the screen-printed electrodes (typically 10%). The precision of the Au electrode structure is higher than that of the AgPd electrode structures, so that the variation in the *R*_25_ value is significantly lower. Taking into account the electrode structure and the corresponding film thickness, a specific resistance in the range of about 65 Ω·m results. The very good reproducibility of the determined *B* value in Figure 4b is worth mentioning. In the as-deposited state, the calculated *B* value is 4250 K with a tolerance range of only ±1% and is not influenced by the electrode material. The slightly higher *B* value scattering with the AgPd electrode is due to measurement inaccuracies.

### 3.2. Film Characterization in the Tempered State—Thermal Stability 

**Figure 5:**
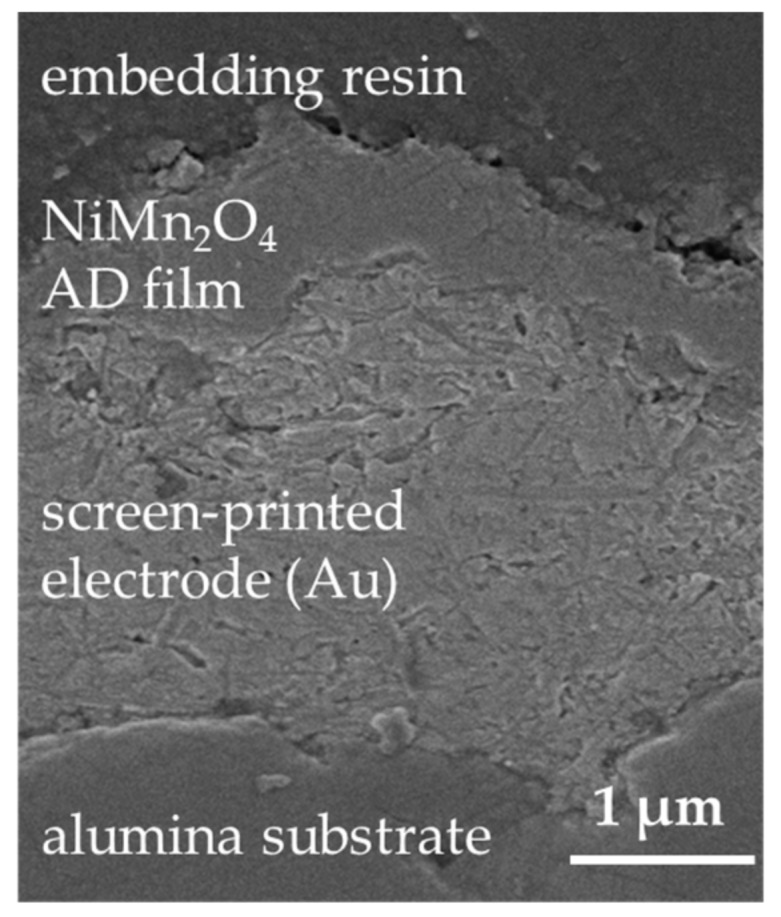
Cross-section of an NTCR component, consisting of a NiMn_2_O_4_ AD film on screen-printed Au electrodes on alumina substrate, after being tempered under cycle C800.

In general, it can be found out that the deposited films on both electrode materials are mechanically stable irrespective of the temperature treatment. No cracks, spalling or delamination could be detected, as shown in Figure 5. It shows the cross-section of a NTCR component, consisting of the NiMn_2_O_4_ film on an Au electrode, after being tempered under cycle C800.

**Figure 6:**
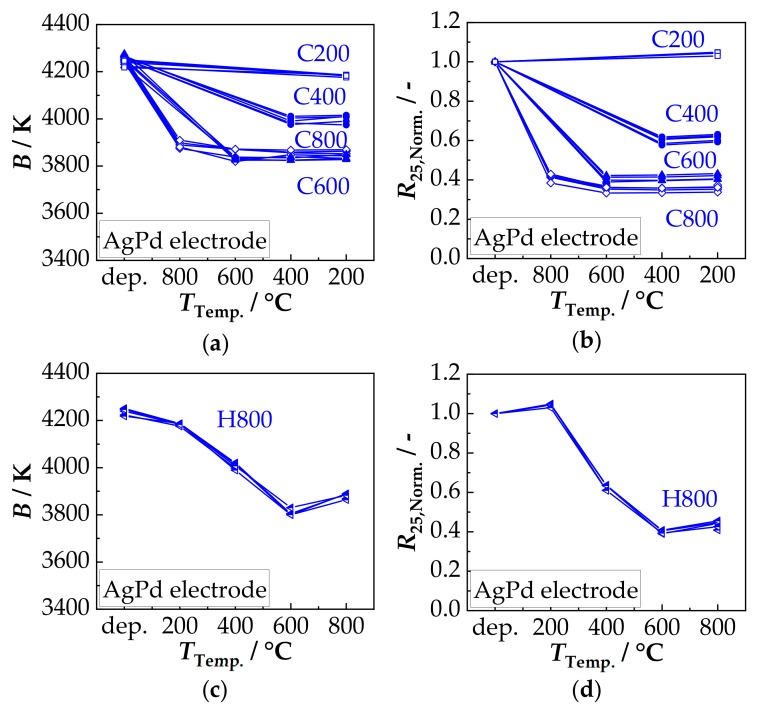
AgPd electrode: Tempering-related change of (**a**) *B* and (**b**) *R*_25,Norm._ in the as-deposited state (dep.) and after cycles C800, C600, C400, and C200, respectively, and of (**c**) *B* and (**d**) *R*_25,Norm._ in the as-deposited state and after cycle H800.

**Figure 7:**
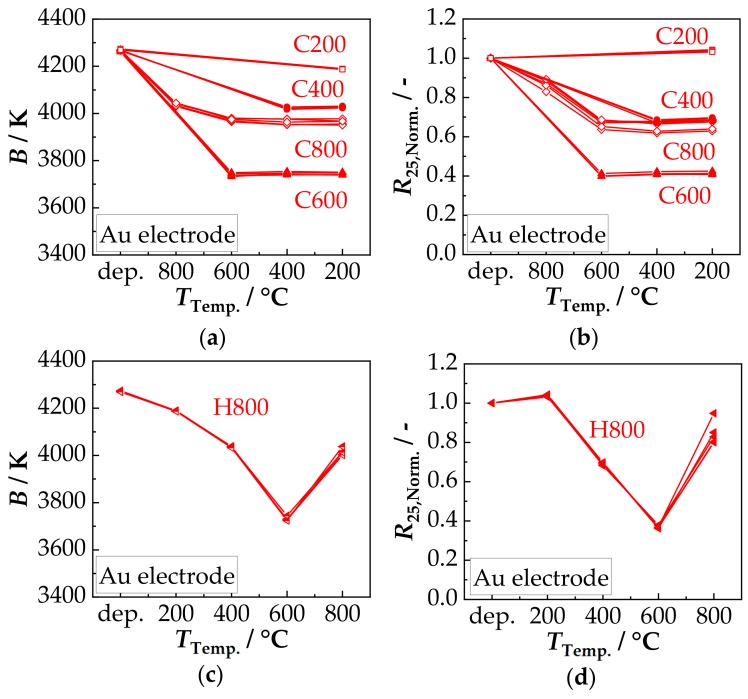
Tempering-related change of (**a**) *B* and (**b**) *R*_25,Norm._ in the as-deposited state (dep.) and after the cycle C800, C600, C400, C200; tempering-related change of (**c**) *B* and (**d**) *R*_25,Norm._ in the as-deposited state and after the cycle H800 of the NTCR devices with Au electrode.

The results from the electrical analysis of the thermal stability are summarized in the following Figure 6 (AgPd electrode) and Figure 7 (Au electrode). Five components per temper cycle (C800, C600, C400, C200 and H800) were tested. From the determined *R*_25_ and *R*_85_ values, the *B* values were calculated according to Equation (3). The *R*_25_ values were normalized to the *R*_25_ values in the as-deposited state.

Figure 6a shows the determined *B* values of cycles C800 to C200. It is shown that after first short-term tempering between 200 °C and 800 °C (60 min), the *B* value, starting from the value of the as-deposited state of 4250 K, is reduced. A minimum of 3830 K is achieved after tempering at 600 °C. While a further reduction occurs in cycle C800 with the second tempering at 600 °C from 3880 K to 3830 K, in cycles C600–C200 the extent of the reduction is determined by the first tempering step (no further change after tempering at 400 °C and 200 °C). If tempered in reverse order (cycle H800, Figure 6c), the behavior is identical. Here, the *B* values decrease as the temperature increase and reach a minimum of 3830 K after tempering at 600 °C, as well. Then, the *B* values increase slightly to 3880 K. As a result, the same *B* values are obtained at the same maximum tempering temperature regardless of the order of tempering (comparison of C800 and H800). The temperature-related change of *R*_25,Norm._ is shown in Figure 6b. Cycle C200 shows a slight increase to 1.03 after being tempered at 200 °C. In cycles C800-C400, the *R*_25,Norm._ falls to 0.4 (C800, C600) or 0.6 (C400) after the first temper treatment. Here too, in cycle C800, the *R*_25,Norm._ changes slightly to 0.35 after the second tempering at 600 °C. All the following temper treatments at 400 °C and 200 °C do not further affect the resistance. A similar behavior occurs in the reverse cycle H800 (Figure 6d). First (*T*_Temp._ 200 °C) the *R*_25,Norm._ rises to 1.04 and then drops to 0.6 (*T*_Temp._ 400 °C) and further to a minimum of 0.4 after tempering at 600 °C. Afterwards, the *R*_25,Norm._ rises again to about 0.45. Consequently, the *R*_25,Norm._ values at the same maximum tempering temperature are approximately the same and are, therefore, independent of the tempering direction (C800 to H800).

In order to investigate the influence of the electrode material on the tempering behavior and on the thermal stability, the same test series was carried out with NTCR components with Au electrode. The results are summarized in Figure 7.

In Figure 7a the tempering-related change in the *B* value of cycles C800–C200 is shown. Starting from the deposited state of 4250 K, the *B* value is reduced as a result of the first short-time tempering. In Cycles C600–C200, the extent of reduction increases with increasing tempering temperature and reaches a minimum of 3740 K after tempering at 600 °C. In the cycle C800, the *B* value after the first tempering at 800 °C is reduced to only 4030 K and is thus in the range of the *B* value of cycle C400. After the second tempering at 600 °C in cycle C800, the *B* value is reduced to 3975 K. All subsequent temper processes at 400 °C and 200 °C do not lead to any further changes in all cycles (C800–C200). The inverse cycle H800 shows the same behavior at the same tempering temperature. This means that the *B* value is reduced by tempering between 200 °C to 600 °C with increasing tempering temperature and reaches a minimum of 3740 K. After tempering at 800 °C, the *B* value rises to about 4030 K. A comparison of the change in *B* values due to tempering between the electrode materials Au and AgPd shows some differences. While at low tempering temperatures (200 °C: both 4185 K, 400 °C: AgPd: 4020 K, Au: 4030 K) little or no difference is observed, the differences are more pronounced at higher tempering temperatures. The difference after tempering at 600 °C is about 90 K (AgPd: 3830 K, Au: 3740 K) and at 800 °C 150 K (AgPd: 3880 K, Au: 4030 K). The tempering-related change of *R*_25,Norm._ is shown in Figure 7b. Similar to the components with AgPd electrode (Figure 6b), *R*_25,Norm._ increase to 1.03 after short-term tempering at 200 °C. In cycles C800–C400, however, *R*_25,Norm._ is reduced after the first tempering step. This is about 0.68 for cycle C400 and 0.4 for cycle C600. In cycle C800, the *R*_25,Norm._ is 0.86 after the first tempering and 0.63–0.68 after the second tempering at 600 °C, thus in the range of cycle C400. All subsequent temper treatments at 400 °C and 200 °C have no further influence on *R*_25,Norm._. In the case of the same tempering temperature, it can also be seen that the values in the inverse cycle H800 are identical to the values after the first tempering in cycles C800–C200. Hence, also for Au electrodes, the order of the temperature treatment has no influence on *R*_25,Norm_. 

**Figure 8:**
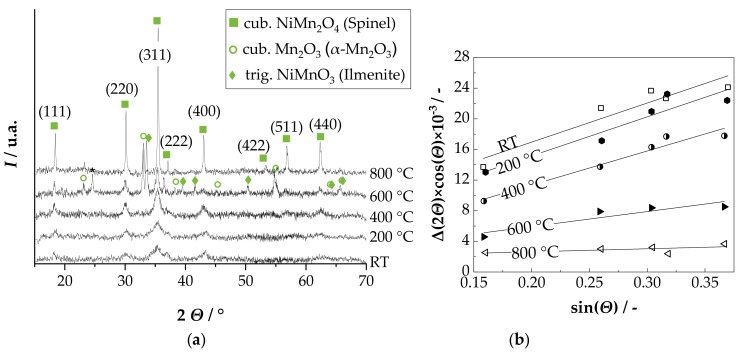
(**a**) XRD pattern of the aerosol deposited NiMn_2_O_4_ film on a Si-wafer analyzed in the as-deposited state and after tempering at 200 °C, 400 °C, 600 °C, and 800 °C, respectively, for 30 min; (**b**) Williamson–Hall plot for all spectra.

The tempering-related change in *R*_25,Norm._ for the two electrode materials (AgPd and Au), are almost the same for C600–C200. However, there are differences in the C800 and H800 cycle, which may be caused by the second tempering step at 600 °C in the C800 cycle. To confirm or exclude cation migrations as the cause for the observed temperature effects on the *B* and *R*_25,Norm._ value, a high temperature XRD measurement at 200 °C, 400 °C, 600 °C, and 800 °C was conducted in air. The obtained XRD patterns are shown in Figure 8a.

In the XRD pattern at room temperature (RT) and after 30 min at 200 °C and 400 °C, only the reflexes of the cubic NiMn_2_O_4_ spinel can be found. First, (RT) reflexes show a strong widening as well as a low intensity, as it is typical for AD films. As the temperature rises from 200 °C to 400 °C, the reflections become narrower and more intense. After 30 min at 600 °C, the reflexes of the cubic Mn_2_O_3_ (α-Mn_2_O_3_) and the trigonal NiMnO_3_ (ilmenite) occur in addition to the cubic NiMn_2_O_4_ spinel. This is in coincidence with the phase diagram by Wickham [44]. In addition, the reflexes are even narrower, with higher intensity. In the XRD pattern at 800 °C, only the reflections of the cubic NiMn_2_O_4_ are found again. These reflexes are the tightest, with the highest intensity. Evaluation of the XRD patterns using the Williamson–Hall method (Figure 8b) shows that the crystallite size increases from 21 nm (RT), over 28 nm (200 °C), 56 nm (400 °C) and 69 nm (600 °C) to 75 nm (800 °C) with increasing temperature and time. At the same time, the internal strain, starting from 1.3% in the as-deposited state (RT) and in the 200 °C tempered state, is reduced to 1.1% in the 400 °C, 0.5% in the 600 °C and 0.1% in the 800 °C tempered state.

### 3.3. Film Characterization in Aged State—Aging Stability 

**Figure 9:**
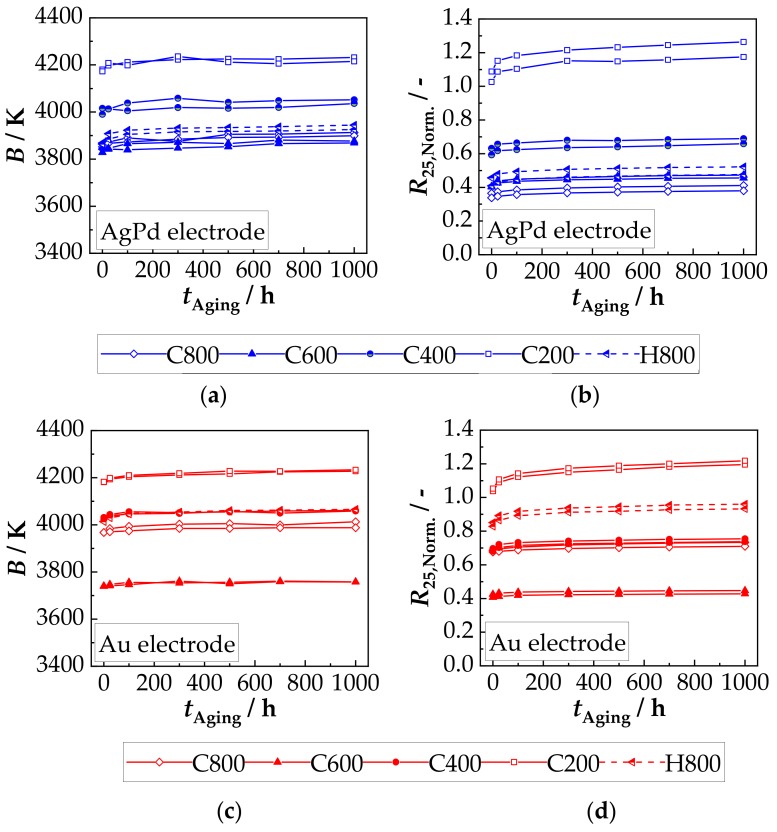
Aging-related change in (**a**) *B* and (**b**) *R*_25,Norm._ of components with AgPd electrodes and (**c**) *B* and (**d**) *R*_25,Norm._ of components with Au electrodes during aging at 125 °C in air.

Aging of components in the as-deposited state leads to very high changes in *B* as well as in the *R*_25_ value. The change for AgPd electrodes after 700 h at 125 °C is +32% for the *R*_25_ value and −2.3% for the *B* value. A similar behavior can be seen for Au electrodes. After aging at 125 °C for 700 h, *R*_25_ increases by +17% and the *B* decreases by −0.95%, compared to the as-deposited state. In order to investigate whether a previous tempering, as described in the previous section, has an influence on the aging behavior, two components were aged at 125 °C for 1000 h per each tempering cycle and per each electrode material. How *B* and *R*_25,Norm._ are affected due to aging for both electrode materials is shown in Figure 9a–d (compared to the tempered state (*t*_Aging_ = 0 h)). 

**Figure 10:**
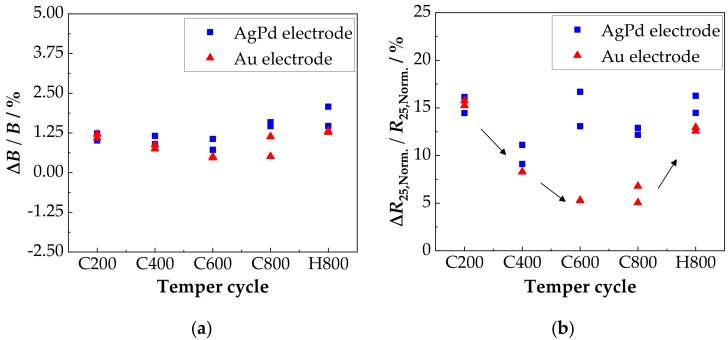
Aging resistance as it depends on a previous temper process: Comparison of the relative change of (**a**) *B* and (**b**) *R*_25,Norm._ relative to the tempered state (*t*_Aging_ = 0) after 1000 h aging at 125 °C in air for the samples deposited on electrode materials AgPd and Au.

It is shown that the main change of *B* and *R*_25,Norm._ occurs within the first 100 h, regardless of the electrode material. In addition, the reduction in *B* and *R*_25,Norm._ achieved by tempering changes only slightly after aging at 125 °C for 1000 h. For better comparability, the percentage change of the *B* and *R*_25,Norm._ after 1000 h aging relative to the starting value (0 h aging corresponds to the tempered state) was calculated and plotted against the maximum tempering temperature in Figure 10.

This shows that the change in *B* after 1000 h of aging at 125 °C is in the range between +0.5% and +1.5%, irrespective of the temperature condition and the electrode material (see Figure 10a). An influence of tempering as well as the electrode material, however, can be seen in the percentage change of the *R*_25,Norm._ (Figure 10b). First, the behavior of both electrode materials is similar. After 1000 h of aging, the change is reduced from about +15% for the 200 °C tempered components (C200) to +9% for the components (C400) tempered at a maximum temperature of 400 °C. Aging of the moderately tempered components is thus already significantly lower than that in the as-deposited state. Significant differences in the aging behavior of the electrode materials can be seen at the maximum tempering temperatures of 600 °C (C600) and 800 °C (C800 and H800). With the AgPd electrode, the aging-related change in *R*_25,Norm._ after a maximum tempering temperature of 600 °C and 800 °C (C600, C800 and H800) increase to about +13% to +15%. A clear influence of the tempering cycle (comparison C800 to H800) is not discernible. With the Au electrode, on the other hand, the percentage change in *R*_25,Norm._ decreases further to about 5.5% at the maximum tempering temperature of 600 °C in cycle C600. While in cycle C800 with the maximum tempering temperature of 800 °C, the percentage change is also in the range of +5.5%, the inverse cycle H800 shows a change of about +12.5%. This difference is in contrast to the AgPd electrode, where the aging after cycles C800 and H800 is approximately equal. Consequently, the electrode material, the maximum tempering temperature as well as the applied tempering cycle have an influence on the aging behavior. Possible reasons for that are discussed in the following section.

## 4. Discussion

In the as-deposited state, the AD film has a crystal structure of a single-phase cubic spinel, since no phase change of the original powder occurs during aerosol deposition. Together with a dense, nanocrystalline film structure with crystallite sizes in the AD-typical range of 20 nm [19,40,41,43] and an excellent adhesion to both the substrate and the electrode, a successful aerosol deposition according to the RTIC mechanism [22] can be assumed. The achieved NTCR characteristics, *B* constant with 4250 K and specific resistance at 25 °C *ρ*_25_ with about 65 Ω·m are independent of the electrode material and well above the typical values of bulk ceramics (3500 K–3900 K and 20 Ω·m–30 Ω·m [4,45,46,47,48]). This can be attributed to several factors: used powder, nano-grained film structure and production-related film strains. In the case of the used powders, the purity of the starting materials and the type of powder preparation (chemical or ceramic route) have a decisive influence on the NTCR characteristics. According to Rousset et al. [48], the *B* value of the bulk ceramic can vary in extreme cases in the range from 3680 K to 4260 K depending on the preparation route and the purity of the starting materials. The *ρ*_25_ value can even vary from 10 Ω·m to 320 Ω·m. In the case of grain sizes in the lower nanometer range, as is typical for AD films, the high proportion of grain boundaries may increase the electrical resistance [18,26,49]. The same applies to the high internal strains. They yield to distortions in the crystal lattice and thus influence the hopping processes of the charge carriers. The study of the tempering behavior confirms the existing observation [15,17,19,20] that both *B* and *R*_25_ are reduced by moderate tempering of AD-NiMn_2_O_4_ films. However, the study shows further effects. In the low temperature range of 200 °C and 400 °C, *B* and *R*_25,Norm._ are reduced after initial tempering. If a new short-term heating in this temperature range takes place, no further change can be seen if the previous temperature treatment was carried out at higher or identical temperatures. This effect is on the one hand, as shown in the XRD analysis, due to a reduction of film strains and crystallite growth. Cation migrations to the thermodynamically preferred place between tetrahedral and octahedral sites from about 300 °C can also occur [50]. However, there is no change in the crystal structure. As described by Tang et al. [51] and proven by the XRD measurements, the cubic spinel is stable. At temperatures above 400 °C, *B* and *R*_25,Norm._ initially drop to a minimum after tempering at 600 °C and then increase again after tempering at 800 °C. There are also significant differences in the behavior of the Au and AgPd electrodes. The reason for this may be the interaction of several effects. As shown in the XRD, the film strains are further reduced and the crystallite sizes are increased. It can also be assumed that the further reduction of *B* and *R*_25,Norm._ is due to cation migration, oxidation in the spinel phase and partial oxidative decomposition of the NiMn_2_O_4_ spinel. They occur in the temperature range up to 730 °C. It is reported that as a result of cation migration (e.g., Ni^2+^ from octahedral to tetrahedral sites), the conductivity increases by 20%–30% [52]. Fritsch et al. [24] describe that this migration-related change in conductivity is further increased by oxidations in the spinel structure. The partial oxidative decomposition, according to NiMn_2_O_4_ + ¼ O_2_ --> NiMnO_3_ + ½ α-Mn_2_O_3_ [53], was detected (Figure 8) by XRD and is in accordance with the phase diagram according to Wickham [44]. α-Mn_2_O_3_ (*E*_A_: 0.6 eV and 10^3^ Ω·m [54]) and NiMnO_3_ (*E*_A_: 0.44 eV and 10^6^ Ω·m [55]) are both comparatively high-ohmic. However, it is possible that spinels with less Ni Ni*_x_*Mn_3-*x*_O_4_ (0.57 < *x* < 1) form during decomposition as well. They cannot be resolved in the XRD pattern due to the reflex broadening. With these spinels, the activation energy *E*_A_ decreases noticeably with decreasing nickel contents to a minimum at *x* = 0.8 with about 0.35 eV [56]. The Ni-poor spinels could therefore also contribute to the reduction of *B* and *R*_25,Norm._. Above 730 °C, the described processes are reversible, which explains the renewed increase of *B* and *R*_25,Norm._ after the thermal treatment at 800 °C. This has also been proven by XRD in Figure 8. 

The different behavior of the AgPd electrode after tempering at 600 °C is supposed to be due to irreversible interactions with the AgPd electrode. It is well-known in “dynamic” Ag/AgO/Pd resistor pastes that during cooling from 850 °C, oxidation/reduction processes occur [57,58,59]. This behavior can also be seen, when heating up AgPd electrodes [60]. The oxidation/reduction process start at 450 °C and are finished at 700 °C. It is that the required oxygen for these processes are taken from the spinel that form a dense, airtight film on top of the electrodes. Hence, the NTCR characteristics should be especially affected in this temperature range, as it is observed in Figure 10b. Looking at the aging behavior, a major change can be seen within the first 100 h. This behavior is typical for NTCR ceramics and has been observed many times [23,27,48]. The as-deposited samples showed large changes in *B* and *R*_25_ values for both Au and AgPd. This is partly due to the high strain in the film, which is released during aging (negative *B* value change). It is also possible that as a result of calcination, oxidation processes may have occurred on the surface and therefore cation vacancies are formed. They are distributed non-homogeneously in the deposited film. As the as-deposited samples age, the cation vacancies are distributed homogeneously over the ceramic as suggested by Groen et al. [26]. This leads to a high change in the *R*_25_ value. The tempered components, on the other hand, exhibit a homogeneous cation vacancies distribution even before aging, leading to a clearly lower aging. With tempered samples, it can be also shown that after ageing the reduction of *B* and *R*_25,Norm._ achieved by tempering is maintained. This is partly due to the fact that the aging temperature is too low for any cation migration. The percentage change after 1000 h aging at 125 °C shows that there are no significant differences in the *B* value. The change in resistance, on the other hand, shows clear differences. The components treated in cycle C400 (*T*_Temp.,max._ 400 °C) show a higher aging stability for both electrode materials than those treated in cycle C200 (*T*_Temp.,max._ 200 °C). This is partly due to the fact that most of the internal strains within the film have already been reduced and it is also known that larger grains show less aging [28]. With the Au electrode, the change of the electrical properties of the components during aging at 125 °C treated in tempering cycles C600 and C800 is approximately the same and is below that of cycle C400. The components treated in cycle H800, on the other hand, show a higher degree of aging. This can be explained by the fact that the cycle H800 cools down from 800 °C for the last time. The spinel is single-phase but probably oxidized [6,24], which makes it more prone to aging than for example in the samples of the C400 cycle. In the C800 cycle, after tempering at 800 °C, a further tempering at 600 °C also takes place (tempering at 400 °C, 200 °C have no further influence, see above). Correspondingly, the samples of the treatment cycle C800 and C600 contain decomposition products (NiMnO_3_ and α-Mn_2_O_3_) as well as presumed spinels with less Ni in addition to the oxidized spinel phase. The spinels with less Ni have not only a lower activation energy but are also less susceptible to aging [24,27]. These facts may explain the better aging behavior. These effects do not occur with the AgPd electrode. The higher aging in cycles C600, C800 and H800 (*T*_Temp.,max_: 600 °C and 800 °C) is due to the interactions with the electrode as described above.

Thus, it can be summarized, that by a subsequent tempering treatment of the film up to 400 °C not only the NTCR parameters (bulk values are reached) but also their aging stability is improved. Tempering temperatures above 400 °C are not considered to be reasonable, as interactions with the electrode as well as oxidations in the spinel phase and partial decomposition may occur.

## 5. Conclusions

It was shown on the present article that mechanically stable NTC thermistors can be produced by means of aerosol deposition. These films are dense and exhibit NTCR characteristics slightly above those of comparable bulk NTCRs due to the AD-typical nanocrystalline film structure and production-induced internal stresses. The investigation of the influence tempering and of the aging behavior have shown that a subsequent temper treatment of the film can improve both the NTCR characteristic and its aging stability. However, temperatures above 400 °C proved unsuitable, as interactions with the electrode and oxidation process in the spinel phase may occur. They have a negative effect on the aging behavior. AD films with multiphase crystal structure, after being tempered at 600 °C, proved to be more stable to aging. However, this is probably more an effect of the spinel composition (Ni*_x_*Mn_3-*x*_O_4_, 0.57 < *x* < 1) and less an improvement of the NiMn_2_O_4_ AD film. Tempering at 400 °C for a short time proved to be optimal, as the bulk characteristics and the highest aging stability were achieved.

## 6. Further Perspective

Based on the results obtained so far, the aerosol deposition method is very promising for the commercial production of NTCR components. In the future, it should be investigated whether improvements are possible by using optimized starting powders. Here, for example, an alternative powder production via the oxalic precursor route or an improved powder composition would be interesting. In addition, it could be investigated whether industrial powders from bulk ceramic production can be used for ADM. This could considerably facilitate the industrial implementation of the ADM process. Studies on long-term stability with protective coatings, which is a typical part of commercial NTCR components, would also be beneficial.
